# A Hematologic Disease Disguised as Cutaneous Candidiasis

**DOI:** 10.7759/cureus.19784

**Published:** 2021-11-21

**Authors:** Joana Couto, Patricia Sobrosa, Ana Afonso, Rosana Maia, Luís P Santos

**Affiliations:** 1 Internal Medicine, Unidade Local de Saúde do Alto Minho, Viana do Castelo, PRT

**Keywords:** langerhans cell histiocytosis(lch), cutaneous candidiasis, skin biopsy, langerhans’cell histiocytosis, hematologic neoplasms

## Abstract

Langerhans cell histiocytosis is a rare hematologic neoplasm with a myeloid origin, which can affect numerous organs, the skin being the second most frequently affected by this disease. In this report, a case of a 44-year-old female, who was intermittently followed due to a suspected persistent cutaneous candidiasis in which a skin biopsy revealed Langerhans cell histiocytosis with immunohistochemistry positive for CD1a and S100 protein, is described. The management of Langerhans cell histiocytosis is difficult because these disorders respond inconsistently to immunosuppressive and chemotherapeutic strategies. The authors present this case to highlight a differential diagnosis of refractory cutaneous candidiasis and raise awareness of the importance of skin biopsy in these cases.

## Introduction

Langerhans cell histiocytosis (LCH) is a hematologic neoplasm, caused by the proliferation of Langerhans cells, derived from immature myeloid dendritic cells of the bone marrow, that express an immunophenotype positive for S100 and CD1a [1-3]. It is a rare disease with an incidence of 5-6 cases per 1,000,000 person-year in children and even rarer in adults with 1-2 cases per 1,000,000 person-year [3,4]. LCH affects patients of all ages although it is more frequent in infants with an average age of 3 years old at diagnosis [2]. LCH in adults appears at an average age of 35 years at diagnosis and is associated with a worst prognosis [3]. While LCH is more common in male children when it comes to adults, the female gender has a slightly higher chance of being affected by this disease. It frequently affects the bone, but it can also involve the skin in 40 percent of cases. Cutaneous involvement may present as scaly papules, vesicles, nodules, tumors with erosion, ulceration, crusting or purpura.

## Case presentation

**Figure 1:**
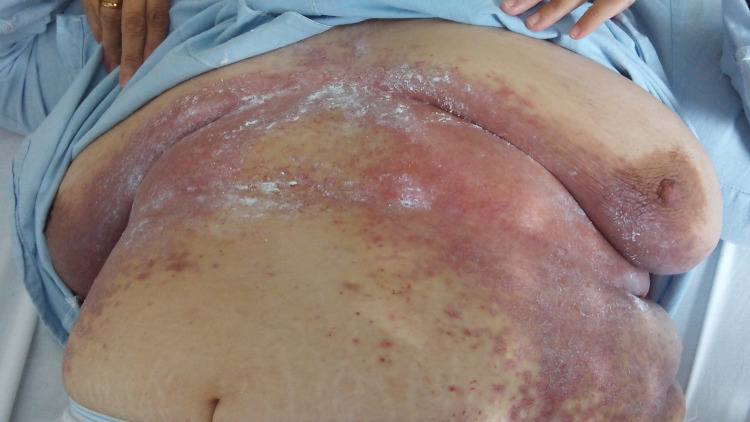
Erosive appearance, with ulcerated vesicles in intertrigo folds.

A 44-year-old female with a history of asthma, essential hypertension, class 3 obesity, depression, and poor social and economic background was intermittently followed during the previous four years for persistent cutaneous candidiasis with intertrigo in the inframammary, inguinal, and lower abdominal regions (Figure 1).

**Figure 2:**
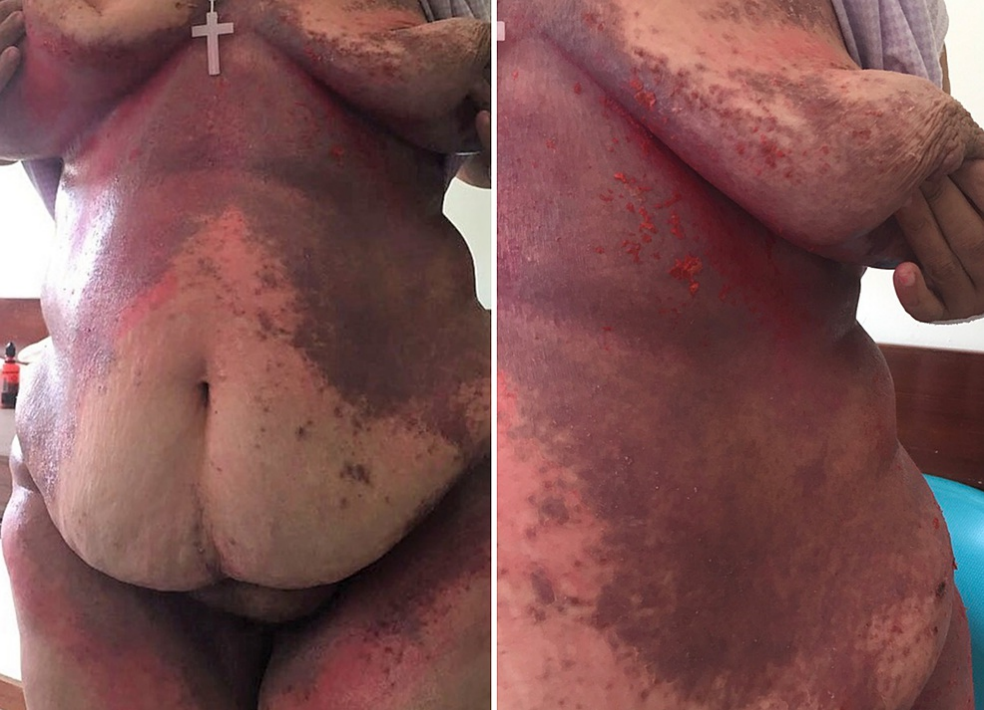
Violaceous papuloscaling and papulocrusted lesions on the torso and intertrigo folds with superficial erosion.

**Figure 3:**
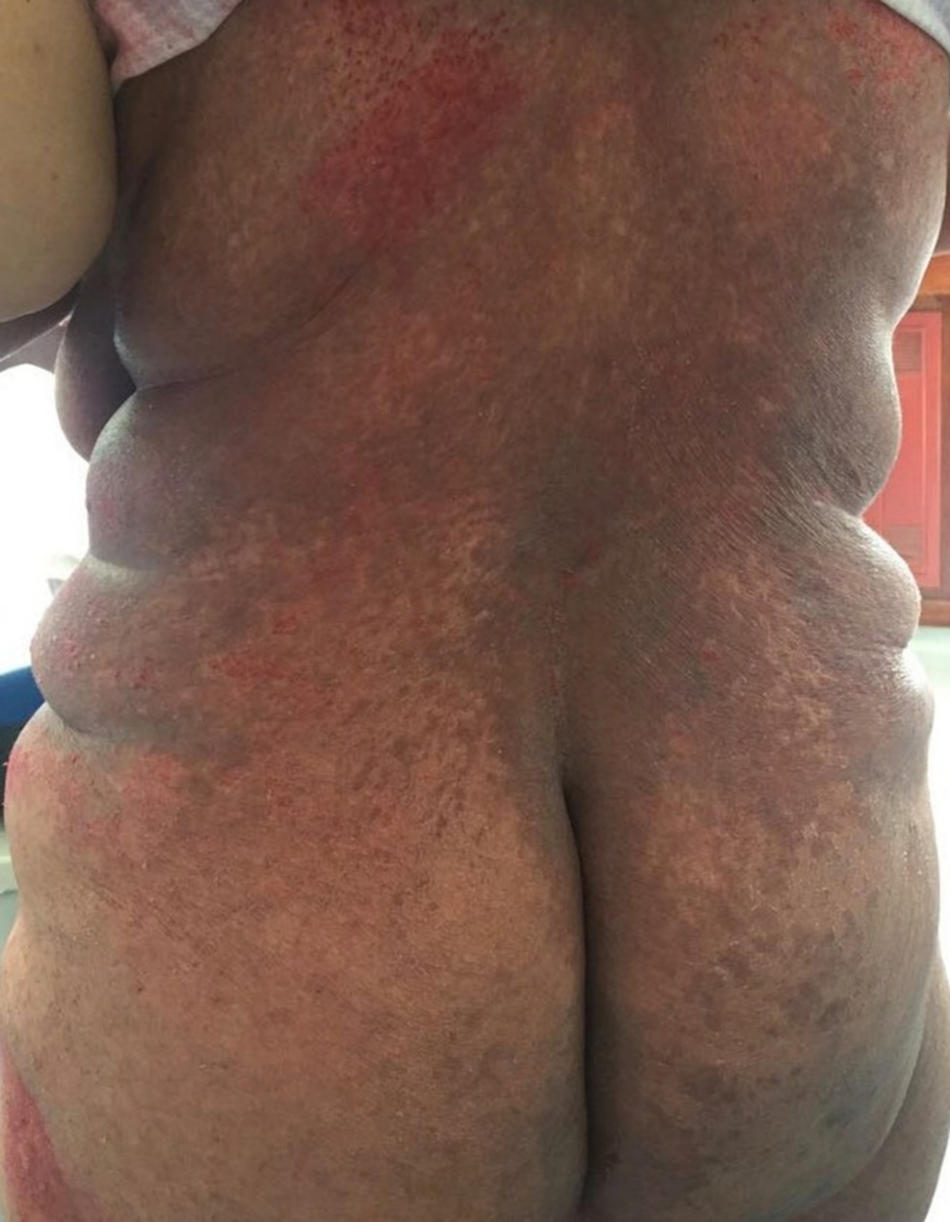
Extensive violaceous ulcerated rash in the back.

**Figure 4:**
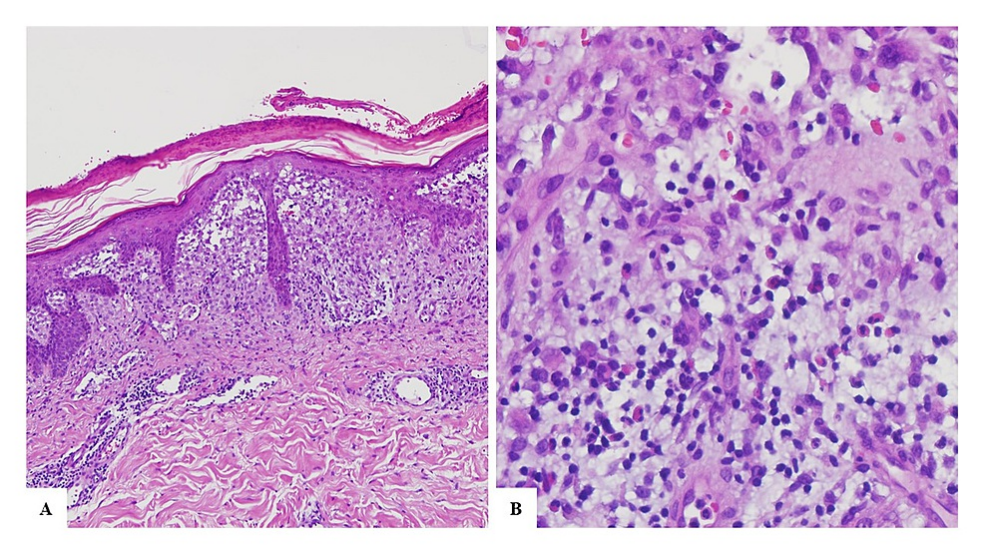
Skin biopsy showing orthokeratotic hyperkeratosis in the epidermis with areas of parakeratosis and infiltrate of cells with eosinophilic cytoplasm and reniform nuclei in the papillary dermis (Hematoxylin and eosin A - x40; B - x400).

**Figure 5:**
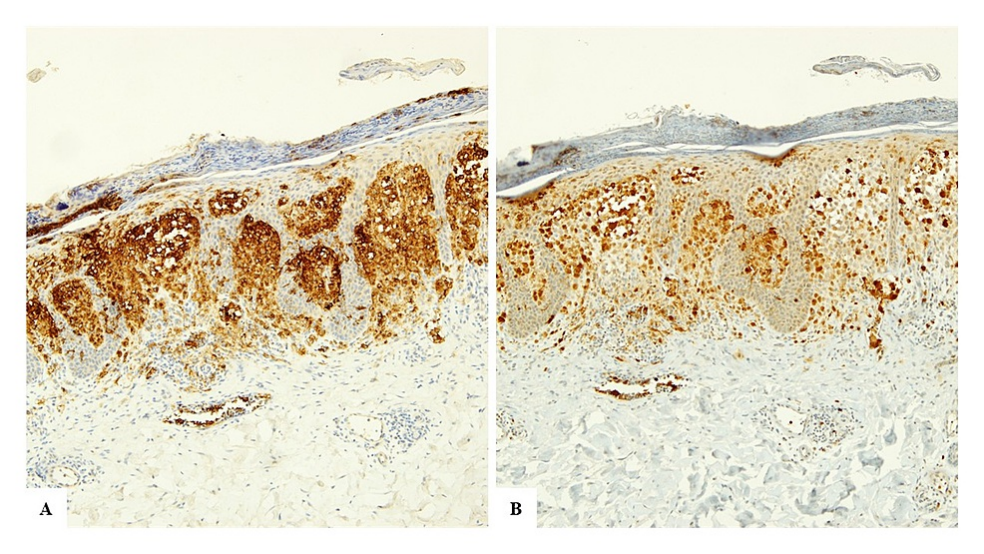
Immunohistochemistry showing positive staining for CD1a (A) and for S100 Protein (B).

She had been treated with topical antifungal, oral fluconazole and oral itraconazole with no improvement, which was believed to be because of poor hygiene and questionable therapeutic compliance. A worsening in the skin rash with exudate, pruritus, and a change to a violaceous colour, with scaly papules and vesicles (Figures 2, 3) led to the performance of a skin biopsy which revealed (Figure 4) orthokeratotic hyperkeratosis in the epidermis with areas of parakeratosis and, in the papillary dermis, there was an infiltrate of cells with eosinophilic cytoplasm and reniform nuclei that showed positive CD1a and S100 proteins on the immunohistochemistry and negative CD163 (Figure 5).

**Figure 6:**
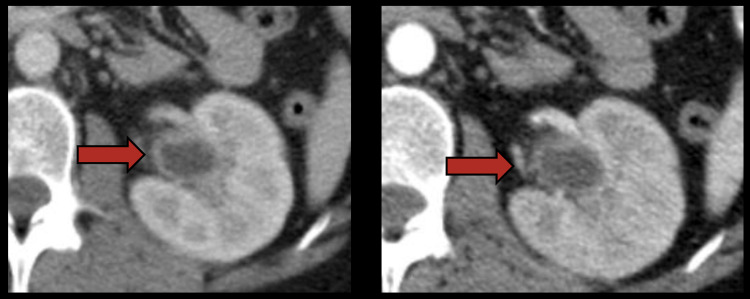
CT showing thickening of the renal pelvis (4 mm) with a slight urothelial dilation (right kidney).

The patient denied constitutional, musculoskeletal, neurological, or urinary complaints. She underwent a complete blood count, complete metabolic panel, brain magnetic resonance imaging (MRI), thoracic-abdominal-pelvic computed tomography (CT), and bone scintigraphy. Brain MRI depicted mild chronic microvascular changes in the white matter, unchanged from a prior study. CT demonstrated a thickening of the renal pelvis (4 mm) in the right kidney with a slight urothelial dilation (Figure 6). The rest of the exams did not reveal further organ involvement.

**Figure 7:**
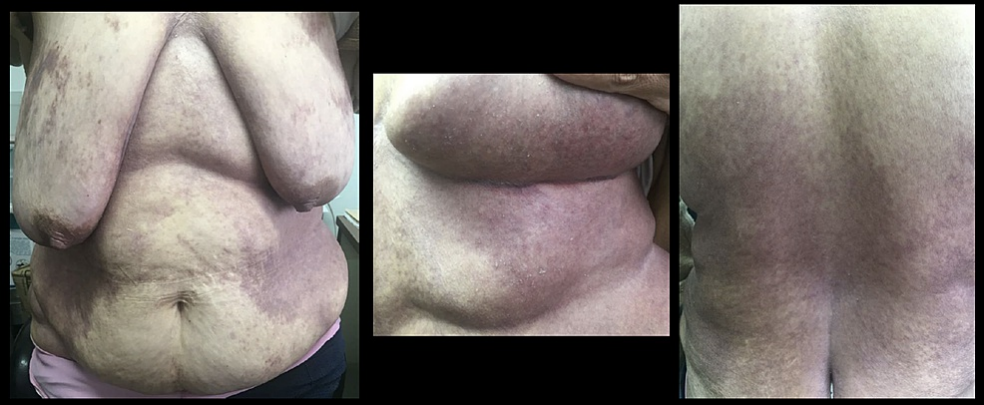
Follow-up after chemotherapy with prednisolone and vinblastine.

After considering the skin histology, the extensive cutaneous involvement, and the infiltrative urothelial involvement, it was evident this was a multi-system process. A consultation with Hematology/Oncology, led to induction treatment with prednisolone and vinblastine-based chemotherapy. At six weeks of chemotherapy, there was a partial regression of the skin lesions (Figure 7) and a resolution of the urothelium lesion in imaging exam (CT).

**Figure 8:**
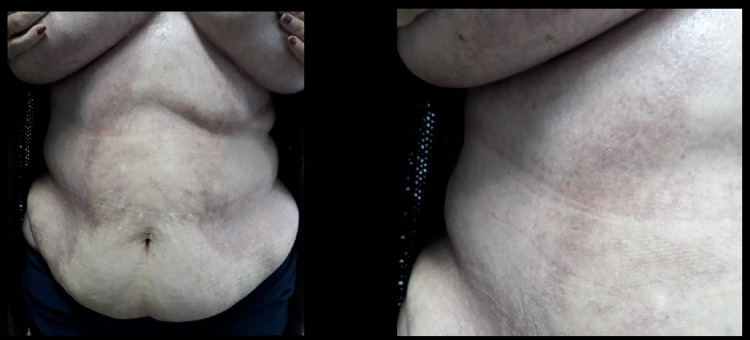
Evaluation after maintenance therapy with mercaptopurine (six months after).

The disease was in continuous regression and considering the extension of affected skin tissue a second round of chemotherapy with prednisolone and vinblastine was administered for six weeks. There was a resolution of all the lesions following this second round, and the patient underwent maintenance therapy consisting of administrating mercaptopurine daily and prednisolone/vinblastine every three weeks during 12 months, staying in remission (Figure 8).

Six months after the end of maintenance therapy the patient had a recurrence of the disease and started second-line chemotherapy with clofarabine and cytosine arabinoside (ARA-C). The patient did not comply with the treatment and the disease progressed. As a result of skin ulceration, she developed skin and soft tissue infection that evolved into septic shock and did not survive despite intensive care support.

## Discussion

Clinical classification of LCH depends on the number of affected organ systems, being divided into single or multi-system diseases. All systems can be affected, but the most frequent is the bone (80% of cases) followed by the skin (about 40%) [2,5]. Isolated cutaneous LCH in adults is rare (2% in a large series) and can present as a single lesion or, more often, as multifocal [4,6]. The most common lesions are squamous papules, sometimes coalescing with seborrheic or eczematous aspect, or red-brown nodules, at times ulcerated [4,6]. The torso, face and scalp are frequently involved as well as large intertrigo folds where the disease assumes an erosive appearance, like in the presented case [4,6]. LCH can mimic seborrheic dermatitis, eczematous dermatitis, candida intertrigo (as observed in our patient), or manifest as solitary or grouped papules, nodules, or ulcerations. Pruritus is a common symptom mentioned by patients [7,8]. Regardless of the primary lesion, LCH presents most often on the torso, head and neck, followed by extremities, intertriginous sites, buttocks, and occasionally the oral mucosa. The gold standard for the diagnosis of cutaneous LCH is skin biopsy with immunohistochemical stains for CD1a, CD207, S100 and birbeck granules on ultrastructural examination [5,9]. LCH natural evolution can range from fulminant disseminated disease to localized bone lesions with a spontaneous resolution [2,4]. No consensus exists for the optimal therapy for LCH particularly in the case of multi-system organ disease. There is no uniform treatment. To decide which would be the best approach to treat each case, the patients are classified into single-system LCH or multi-system LCH, local or multifocal, and with or without the involvement of risk organs [1,2,5]. Regarding cutaneous LCH, this type of single-system can be treated with topical nitrogen mustard, topical corticosteroids, topical tacrolimus, oral methotrexate, or phototherapy whereas multi-system is usually treated with vinblastine-based chemotherapy, as it was decided in this case [1,6,9]. All patients should undergo a thorough history and physical examination to assess the involvement of the disease [10]. The following organs should be evaluated: skin, lymph nodes, ears, oral cavity and mucosa, skeletal system, lungs, thyroid, liver, spleen, and central nervous system [8]. Constitutional symptoms as fever, chills, fatigue, weight loss, lymphadenopathy, polyuria and arthralgia should be enquired, as they may indicate bone marrow, lymph node or pituitary involvement [7]. A complete blood count, comprehensive metabolic panel, skeletal survey, chest radiography and sonography of the liver and spleen are recommended, bone scintigraphy and bone marrow biopsy should also be considered before starting treatment [8,10,11]. All patients with single and multi-system disease are at risk for both local and distant recurrence. Thus, these patients should be carefully examined for evidence of the disease in other organs or for the possibility of recurrence [12]. The probability of disease reactivation within five years of achieving a first complete disease resolution is 46 percent, most of which occur within the first two years [13].

## Conclusions

This case aspires to raise awareness of cutaneous LCH, a possible differential diagnosis of cutaneous candidiasis. The importance of a skin biopsy is showcased in the evolution of this patient. It is important to adequately screen all patients diagnosed with cutaneous LCH for systemic manifestations of this disease, as this can affect management and overall prognosis.
